# Increased receptor affinity of SARS-CoV-2: a new immune escape mechanism

**DOI:** 10.1038/s41541-022-00479-9

**Published:** 2022-05-25

**Authors:** Martin F. Bachmann, Mona O. Mohsen, Daniel E. Speiser

**Affiliations:** University Hospital and University of Bern, Bern, Switzerland

**Keywords:** Viral infection, Epidemiology

## Abstract

‘Affinity escape’: Novel SARS-CoV-2 variants may escape immunity by raising the RBD-ACE2 affinity high enough to outcompete the avidity of neutralizing antibodies.

SARS-CoV-2 has proven to be a rapidly evolving RNA virus with steadily emerging new viral variants. Several of them show enhanced infectivity and/or escape from neutralization by monoclonal antibodies (mAbs), and some were termed variants of concern (VoC).

Extended escape from protective antibodies is well described in virology as so-called serotype formation^1^. Serotypes avoid recognition by previously generated anti-viral antibodies because they differ in neutralizing epitopes due to viral mutation and selection. Typical examples of such serotype-forming viruses are Polio and Dengue viruses, forming 3 and 4 distinct and stable serotypes, respectively^1^.

For the Beta and Gamma variants of SARS-CoV-2, it has recently been shown that the E484K mutation within the receptor binding domain (RBD) of the spike protein strongly reduces recognition by convalescent sera from individuals after infection with the original Wuhan strain^2,3^. While this observation suggests development of viral serotypes, it has been difficult to make the reverse observation, i.e., to find or induce sera that neutralize variants with the E484K mutation better than the Wuhan strain^3–5^. Thus, the current data do not fully demonstrate the existence of different SARS-CoV-2 serotypes. Generally, the demonstration of mAbs capable of distinguishing distinct variants is not sufficient to declare new serotypes, as serotypes are defined by polyclonal anti-sera. In contrast, we have recently demonstrated that viral strains can escape neutralizing antibodies despite essentially preserved recognition specificity: by ways of increased receptor affinity. While RBD of the Wuhan strain binds ACE2 with an affinity of 2–10^−8^ M, RBD with the L452R and E484Q mutations (such as in the Delta and Kappa variants) shows a 4–5 fold higher affinity for the receptor^3^. This not only causes the well-known increased infectivity but also shifts the virus-receptor equilibrium to the right, i.e., the virus binds more strongly to the ACE2 receptor. Indeed, neutralizing antibodies after infection and vaccination have difficulties in competing with RBD-ACE2 binding, suggesting that neutralization is reduced despite that the epitopes of the mutated RBD maintained essentially the same specificity for these antibodies (Fig. 1). Thus, increasing receptor affinity may constitute a new pathway for viruses to escape neutralizing antibodies and may be called affinity escape^6^. Differences in assay setups (e.g., plate coating of ACE2^7^ versus coating of RBD on sensor chips^3^) may lead to different affinities measured in specific cases. Nevertheless, it is well documented that some VoCs exhibit clearly higher affinities for ACE2^3,7–9^, supporting the notion that the above-described affinity type escape is a general phenomenon of SARS-CoV-2.

**Fig. 1:**
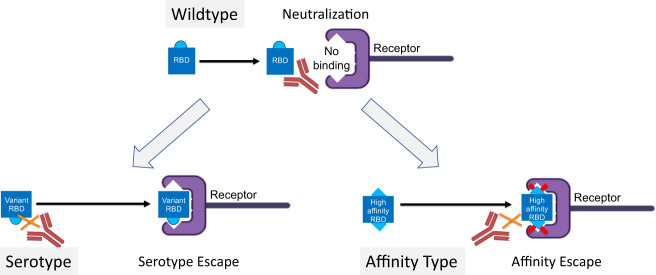
Serotype escape or Affinity escape. Top: Neutralization of wildtype virus by pre-existing antibodies that block RBD binding to the receptor on the surface of susceptible host cells. Bottom left: RBD mutations may alter B cell epitopes such that pre-existing antibodies can no longer neutralize (“Serotype escape”). Bottom right: Alternatively, immune escape may occur because RBD affinity has increased to the extent that the binding of pre-existing antibodies is too weak for competing with RBD-receptor binding. Therefore, the antibodies are unable to neutralize the virus (“Affinity escape”). RBD, receptor binding domain.

Consequently, vaccine optimization should aim at inducing higher avidity antibodies, rather than altered epitope specificity as would be required for new serotypes^10^. Indeed, Wuhan strain-based vaccines that induce high avidity antibodies also protect from severe disease caused by prominent VoCs^3^. Even protection from the Omicron variant is achieved to a relatively high degree after three vaccinations^11^, likely because this induces high titers of antibodies with increased avidity. The latter is promoted by the well-known mechanism of antibody avidity maturation which is enhanced by booster vaccination and with time^12^. Interestingly, booster vaccination after a prolonged interval to the first vaccine dose resulted in enhanced antibody responses^13^. Importantly, a longer vaccination-infection interval was found to be associated with increased neutralization potency to Omicron after breakthrough infection^14^, an observation consistent with the notion that protection from variants may be favored by progressive affinity maturation during the intervals. Re-infection by VoCs and SARS-CoV-2 in general is favored by the fact that the avidity of antibodies induced by natural infection is relatively low^15,16^, probably related to the low capability of SARS-CoV-2 to induce neutralizing antibodies^17^ and the poor formation of germinal centers required for antibody avidity maturation^12,18^. mAb therapy of infection with viral variants is improved by co-administration of multiple neutralizing mAbs with several different relevant specificities^19^, reflecting the superiority of polyclonal as opposed to monoclonal antibodies and the presence of multiple neutralizing epitopes on the spike protein and its RBD^20^. In comparison to mAb therapy that can only be done with a low number of different antibodies, serum antibodies have the advantage of being highly polyclonal and multi-specific.

Evolutionary, novel serotypes are unlikely to emerge from viruses that are inefficient at inducing neutralizing antibodies^17^. Instead, such viruses may get bigger selective advantage by increasing receptor affinity. These observations imply that the current large efforts to identify correlates of protection^21^ should include the assessment of antibody avidity^22^ which is often not done^23^. It will be interesting to determine whether Coronaviruses are unique in this regard, or whether similar observations may be made for other viruses with well-known receptors, such as Adenoviruses.

## Reporting summary

Further information on research design is available in the Nature Research Reporting Summary linked to this article.

## Supplementary information

Reporting Summary
